# Immune Response to an Inactivated Vaccine of SARS-CoV-2 (CoronaVac) in an Indigenous Brazilian Population: A Cohort Study

**DOI:** 10.3390/vaccines12040402

**Published:** 2024-04-10

**Authors:** Laís Albuquerque de Oliveira, Isa Rita Brito de Morais, Marcelo dos Santos Barbosa, Silvana Beutinger Marchioro, Layla Oliveira Campos Leite Machado, Michele Ferreira Marques, Tiago da Silva Ferreira, Gabriel Barroso de Almeida, Dyjaene de Oliveira Barbosa, Alex José Leite Torres, Simone Simionatto

**Affiliations:** 1 Health Science Research Laboratory, Federal University of Grande Dourados, Dourados 79804-970, MS, Brazil; lais.alboliv@gmail.com (L.A.d.O.); marcelo_medvet@outlook.com (M.d.S.B.); laylaleite@hotmail.com (L.O.C.L.M.); michelly.marques22@gmail.com (M.F.M.); tiago201609@gmail.com (T.d.S.F.); 2 Laboratory of Immunology and Molecular Biology, Institute of Health Sciences, Federal University of Bahia, Salvador 40170-110, BA, Brazil; isa.ritabm@gmail.com (I.R.B.d.M.); silmarchioro@hotmail.com (S.B.M.); gabrieldbarroso@gmail.com (G.B.d.A.); dyjaene@hotmail.com (D.d.O.B.); ajltorres@gmail.com (A.J.L.T.)

**Keywords:** SARS-CoV-2, vaccine, immune response, CoronaVac

## Abstract

Introduction: Although the adaptive immune responses to the CoronaVac vaccine are known, their dynamics in indigenous communities remain unclear. In this study, we assessed the humoral and cellular immune responses to CoronaVac (Sinovac Biotech Life Sciences, 2021 NCT05225285, Beijing, China), in immunized Brazilian indigenous individuals. Methods: We conducted a prospective cohort study on indigenous Brazilian people between February 2021 and June 2021. Analyses of immune responses were carried out before (T1) and after a vaccination schedule was completed (T2). Demographic data were collected using a questionnaire. Results: We initially included 328 patients; among them, 120 (36.6%) had no SARS-CoV-2 antibodies. Peripheral blood mononuclear cells (PBMCs) were collected from 106 patients during follow-up visits, of which 91 samples were analyzed by immunophenotyping assay to detect SARS-CoV-2-specific memory T-cell response. Post-vaccination, the levels of memory B-cells and Natural Killer T-lymphocytes increased. Bororó village residents, females, and Terena ethnic group members had higher levels of anti-spike IgG antibodies post-vaccination, whereas alcohol and tobacco users had lower concentrations. Conclusions: To our best knowledge, this was the first comprehensive assessment of antibody and T-cell responses against CoronaVac vaccination in indigenous patients. Our findings showed that antibody response and T-cell immunity against SARS-CoV-2 were present in most patients following the vaccination schedule.

## 1. Background

The coronavirus disease 2019 (COVID-19) emerged rapidly, leading to a dramatic increase in the number of infections worldwide. Due to its high transmission rate, by March 2023, COVID-19 had caused more than 6.8 million deaths and infected more than 760 million people around the world [1]. Herd immunity through vaccination is essential to reduce the effects of the COVID-19 pandemic [2]. For this reason, the World Health Organization (WHO) authorized 10 vaccines for emergency use in the global immunization effort [3].

The CoronaVac vaccine was one of the most widely distributed in the world; it is a two-dose vaccine and has an interval of two to four weeks between the doses [4]. Cellular and humoral immunological aspects of CoronaVac underwent various tests and clinical studies, involving several populations and subpopulations [5]. The tested individuals included those with rheumatic and autoimmune diseases, pregnant women, transplant recipients, cancer patients, and individuals with prior COVID-19, among others. These trials provided valuable information on CoronaVac and inactivated virus vaccines in general [6,7,8,9,10]. Although several populations and subpopulations were included in different studies for various vaccines around the world, none included indigenous populations [11]. The Brazilian indigenous population exhibits significant ethnic diversity (among the largest in the world), with over 1.000.000 individuals distributed among 230 ethnic groups [12].

The CoronaVac vaccination of indigenous Brazilians plays a fundamental role in the national immunization strategy against COVID-19. This population in Brazil faces specific challenges due to their ethnic diversity, specific cultural practices, and socioeconomic conditions on their lands [13]. The recognition of the vulnerability of these communities to SARS-CoV-2 infection motivated the Brazilian government to prioritize the vaccination of these groups, raising the need for adapted approaches to deal with their particularities. 

The choice of CoronaVac for this purpose stands out for its wide distribution and efficacy, demonstrated in clinical studies, making it a crucial tool in protecting these communities against the devastating impacts of the pandemic [14,15]. In this study, we characterized the specific humoral and cellular immune responses from indigenous people following vaccination with CoronaVac.

## 2. Methods

### 2.1. Study Design and Population

**Figure 1:**
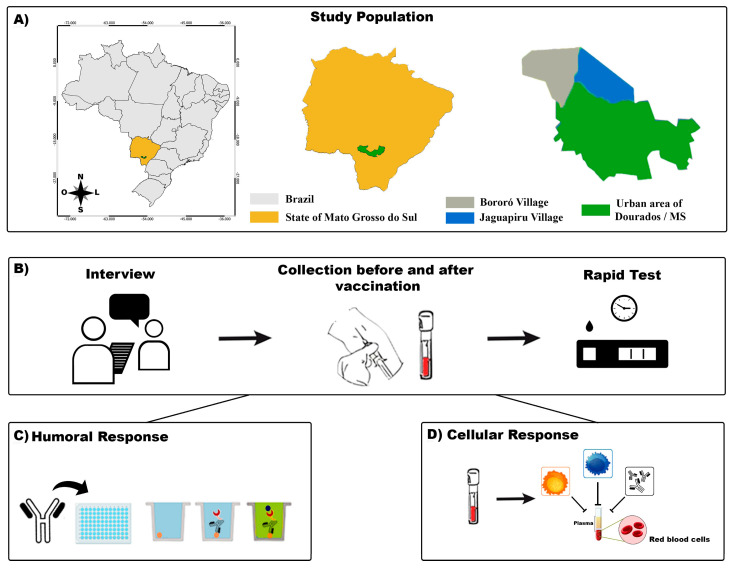
A flowchart of the study. (**A**): The indigenous area in the municipality of Dourados–Mato Grosso do Sul (MS) and its location in the country are shown. (**B**): The participants were interviewed, and their blood samples were collected for SARS-CoV-2 rapid test screening. (**C**): Humoral immunoassay. (**D**): Cellular immunoassay.

Mato Grosso do Sul (MS) shares its borders with Paraguay and Bolivia and has an large Brazilian indigenous population (Figure 1). We performed a cohort study of the indigenous population between February 2021 and June 2021 with participants from the biggest peri-urban Brazilian area [16]. The eligibility criteria were as follows: at least 18 years old, residing in the indigenous area of Dourados-MS, and administered two doses of the CoronaVac vaccine (Sinovac Biotech Life Sciences, 2021 NCT05225285, Beijing, China). We excluded participants with a probable or confirmed SARS-CoV-2 infection, as determined by reverse-transcriptase polymerase chain reaction (RT-PCR) assays or reactive serological testing, and those who were administered only one dose of the CoronaVac vaccine. Written informed consent was obtained from all participants.

### 2.2. Data Collection Procedures

The study was divided into two phases, and blood samples were collected to measure specific humoral and cellular responses at two different times. The first phase of collection (or time 1-T1) was performed before the participants were vaccinated with CoronaVac. The second phase (or time 2-T2) was 45 days after the complete vaccination schedule (two doses of the vaccine). In both phases, blood samples were collected by a professional. After taking appropriate antiseptic measures, 4.5 mL of peripheral venous blood was collected using a vacuum tube system (Figure 1). Peripheral blood mononuclear cell (PBMC) samples were processed to perform cellular immune analysis.

### 2.3. Screening Serological Test

The Leccurate COVID-Antibody rapid test kit (Lepu Medical Technology Leccurate SARS-CoV-2, Beijing, China) was used to detect IgM and IgG antibodies against SARS-CoV-2 for the screening process. Following the manufacturer’s instructions, 10 µL of whole blood was placed in the test plate cavity and ~100 µL of diluent was immediately added. The test was interpreted 15 min after the reaction according to the kit’s instructions. Seropositive participants were excluded from the study. All participants received the results of the rapid test conducted before vaccination.

### 2.4. Enzyme-Linked Immunosorbent Assay (ELISA)

Samples collected in the T1 phase underwent enzyme-linked immunosorbent assay (ELISA) to detect total IgG and IgM antibodies against SARS-CoV-2 (Euroimmun, Pegnitz, Germany). Anti-SARS-CoV-2 QuantiVac™ IgG ELISA (Euroimmun, Germany) was performed with post-vaccination samples (T2 phase) to detect antibodies against the spike protein of SARS-CoV-2. The ELISA tests were conducted using an indirect semi-quantitative method. Serum samples were diluted at a ratio of 1:100 (10 µL of the serum to 1 mL of buffer provided by the kit). The optical density was measured at 450 nm using a spectrophotometer (Multiskan FC, Thermo Scientific, Waltham, MA, USA). The positivity of the ELISA was determined by the cut-off formula provided by the manufacturer. The cut-off points were determined by calculating the ratio between the optical density (OD) values from the control and the OD of the calibrator, following the formula below: OD of control or serum sample/OD of calibrator = ratio. Samples with ratios below 0.8 were categorized as negative, while those with values of 1.1 or higher were considered positive. Results outside this analytical range were re-evaluated with an additional dilution of 1:400. Ratios between 0.8 and 1.1 were considered borderline; these were subjected to additional testing in good time to ensure reliable results. After this, the results were then classified as positive or negative.

### 2.5. Immunophenotyping Assay and Gating Strategy

To conduct the immunophenotyping assays, the peripheral blood mononuclear cells (PBMCs) were isolated by centrifugation at 3.000 rpm for 30 min under a Histopaque separation gradient (sterile-filtered, density: 2.000 g/mL Sigma Aldrich^®^, Burlington, MA, USA). The cells were washed once with an erythrocyte lysis buffer (ACK Lysing Buffer-Gibco^TM^ A1049201, Grand Island, NY, USA) and once with PBS (1600 rpm, 10 min). Finally, the cells obtained were stained with Trypan Blue dye (0.4%) (Gibco^TM^ Grand Island, NY, USA). After counting, the cells were cryopreserved at –80 °C.

To determine cellular profiles via surface and cytoplasmic receptors, immunophenotyping was conducted via eight-color FACSCanto flow cytometry (Becton Dickinson Company, Franklin Lakes, NJ, USA) using monoclonal antibodies labeled with fluorescein isothiocyanate (FITC), phycoerythrin (PE), and PerCP fluorophores. The panel of monoclonal antibodies used is listed in Supplementary Table S1. All monoclonal antibodies were manufactured by Becton Dickinson. Each cell type was processed in one tube and monoclonal antibodies were added and incubated with 100 µL of PBMCs after the FACS lysing buffer was added to eliminate the erythrocytes of the samples, following basic flow cytometry protocols. Samples were acquired from around 100,000 events and analyzed in FACSCanto (Becton Dickinson Company, Franklin Lakes, NJ, USA) using FACSDiva software (version 6.1.3). For the gating strategy, we first selected singular events and excluded doublets. Next, we selected positive events for the pan-leukocyte marker CD45+, which was used as a parameter to identify different cell populations according to the identified receptors.

### 2.6. Data Management and Statistical Analysis

The questionnaire data and the results of SARS-CoV-2 vaccination underwent double registration and were later uploaded to the Research Electronic Data Capture (REDCap) software (version REDCap 8.11.0, Vanderbilt University, Nashville, TN, USA). SPSS 2.7 software (NC, USA) was used to analyze sociodemographic data. Univariate odds ratios were calculated based on 2 × 2 contingency tables to obtain the Odds Ratio (OR) and 95% CI. *p*-Values were calculated using Pearson’s chi-square test or Fisher’s exact test. For the multinomial variables “Ethnicity”, “Schooling”, and “Residents per household”, the OR was calculated based on the reference category (the category in which the OR and *p*-value appear). To assess the difference between the mean values before and after vaccination, GraphPad Prism v.7.0 (San Diego, CA, USA) was used. The means of the triplicates obtained in the ELISA were used. As the data did not follow a normal distribution, the Wilcoxon two-way non-parametric test was performed. The significance level for all analyses was set at <0.053. After performing the Wilcoxon test to compare the means between the groups at different times (T1 and T2), we obtained the *p*-values for each comparison. To manage the type I error rate associated with multiple comparisons, Dunn’s correction was applied to assess the cellular immune response at T1 and T2, using the formula αeu = c × (c − 1)/2α.

## 3. Results

### 3.1. Study Design

Of the 328 indigenous people invited, 120 (36.6%) showed an absence of SARS-CoV-2 antibodies in rapid tests. Among them, 106 participants had their PBMC analyzed, of which 85.84% (91/106) completed the CoronaVac vaccination schedule and were included in the study. The participants had an average age of 36 years, 78% were women, 70% depended on government benefits, 32% used tobacco, 20% consumed alcohol, and 93% had undergone influenza vaccination (Table 1).

### 3.2. SARS-CoV-2-Specific Humoral Responses

**Figure 2:**
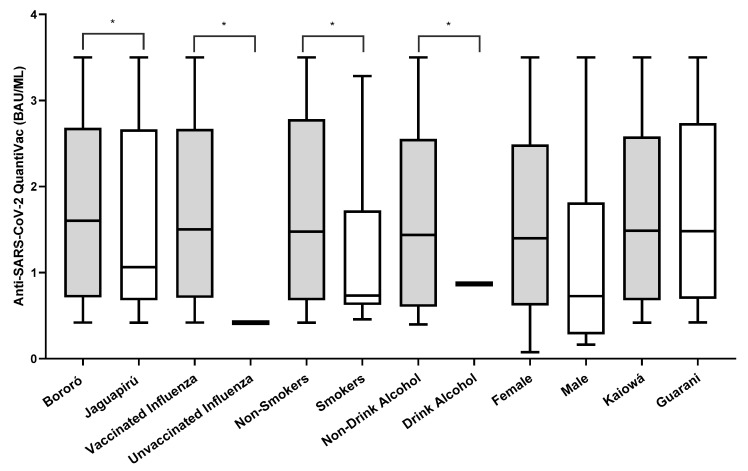
Number of IgG anti-spike antibody titers (BAU/mL) after two doses of the CoronaVac vaccine. * Variables that were statistically significant with *p* < 0.05. Graphing was performed using GraphPad Prism v.7.0 software (San Diego, CA, USA), employing the non-parametric two-way Wilcoxon test. The *p*-values were obtained after Dunn’s correction.

Out of 106 participants negative on the rapid test, 78.03% (83/106) were positive when tested in an anti-NP ELISA. Of those, 2.40% (2/83) had IgM antibodies, 12.04% (10/83) had IgM and IgG, and 85.54% (71/83) had IgG antibodies detected. After the full vaccination schedule, the anti-spike IgG ELISA positivity rate was 67.03% (61/91). The IgG antibody titers against the spike protein (BAU/mL) after vaccination were higher in Bororó village (3.945 BAU/mL vs. 3.865 BAU/mL), participants vaccinated against influenza (4.124 BAU/mL vs. 0.270 BAU/mL), non-smokers (2.806 BAU/mL vs. 1.488 BAU/mL), non-alcohol consumers (2.781 BAU/mL vs. 1.078 BAU/mL), female participants (3.578 BAU/mL vs. 1.793 BAU/mL), and the Guarani ethnic group (3.698 BAU/mL vs. 3.361 BAU/mL) (Figure 2). The univariate odds ratios (OR) indicated significantly lower concentrations of IgG antibodies against the SARS-CoV-2 spike protein among participants who reported being alcoholics and tobacco users. Additionally, participants who were administered the influenza virus vaccine showed significantly higher concentrations of IgG antibodies (Table 1).

### 3.3. Cellular Response

**Figure 3:**
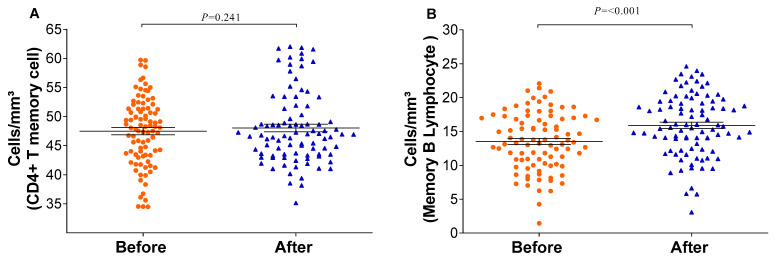
Memory immune response before and after immunization: (**A**) CD4 T-lymphocytes and (**B**) memory B-lymphocytes were quantified by flow cytometry. The graph was created using GraphPad Prism software v.7.0 (San Diego, CA, USA), employing the two-way Wilcoxon non-parametric test. All differences were statistically significant at *p* < 0.05. The *p*-values were obtained after Dunn’s correction.

**Figure 4:**
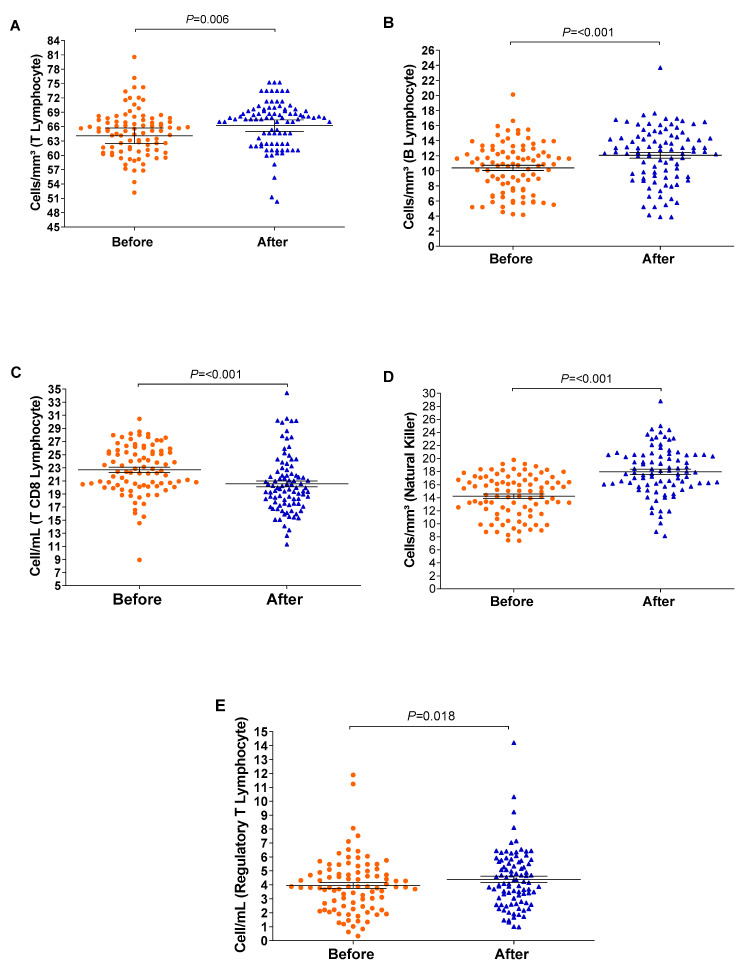
The boxplots show cellular measurements determined by conducting flow cytometry assays; significant changes were recorded before and after vaccination. Measurements of (**A**) T-lymphocytes, (**B**) B-lymphocytes, (**C**) CD8 T-lymphocytes, (**D**) Natural Killer T-lymphocytes, and (**E**) regulatory T-cells. The vertical axis represents the cell count, and the horizontal axis represents the data collected before and after vaccination. The graph was created using GraphPad Prism software v.7.0 (San Diego, CA, USA), employing the two-way Wilcoxon non-parametric test. The *p*-values were obtained after Dunn’s correction.

The relative counts of memory CD4+ T-cells and memory B-lymphocytes were stratified and compared before and after the vaccination schedule with CoronaVac. Forty-five days after completing the vaccination schedule, an increase in the number of T-lymphocytes and B-lymphocytes was observed (Figure 3). Additionally, the proportion of Natural Killer T-lymphocytes and regulatory T-cells increased significantly (*p* = <0.001/0.003). Although the relative count of total T-lymphocytes increased, a reduction in the number of CD8+ T-lymphocytes was observed 45 days after completing the vaccination schedule. Meanwhile, the counts of monocytes and CD4+ T-lymphocytes remained stable (Figure 4). In individuals previously exposed to the virus, there was no significant increase in the immune response, except for the CD4+ T-lymphocytes (*p* = 0.0187*) after the vaccination schedule (Supplementary Table S1).

## 4. Discussion

In this study, we assessed the humoral and cellular immune responses of indigenous people following their vaccination with CoronaVac. Most participants were female, young, and earning less than USD 500. The predominantly female participation not only reflects receptivity of this gender, but also supports findings from previous studies [17,18]. In addition, the home vaccination of the elderly, due to their vulnerability and mobility limitations, justifies the higher prevalence of younger individuals in our study. This trend is particularly evident in indigenous territories, where restrictions make specialized care difficult [19].

Prior to vaccination, ELISA was employed to identify total antibodies against SARS-CoV-2 [20]. The results showed that 78% (83/106) of the population analyzed had anti-NP antibodies, highlighting previous exposure to the virus. Conversely, post-vaccination, ELISA was utilized to assess anti-spike antibodies [21], revealing a positivity rate of 67.03%. These results are similar to already reports for no-indigenous populations [4], providing valuable insights into the immune response elicited by the CoronaVac vaccine within a distinct and underserved demographic. On the other hand, previous infection did not appear to significantly influence the immune response, as there was no notable increase in immune response following vaccination in individuals previously exposed to SARS-CoV-2. It is important to note that anti-NP antibodies do not ensure long-lasting immunity or complete protection against reinfection [22]. The immune response to SARS-CoV-2 seems to be complex, and factors such as neutralizing antibodies and cellular response are crucial role in protection against the disease [23,24].

In addition to assessing the social vulnerability of this population associated with lifestyle factors and the immune response to the vaccine, we also assessed the use of alcohol and tobacco in this study. Alcohol and tobacco users had significantly lower concentrations of IgG antibodies against the SARS-CoV-2 spike protein compared to non-users. Alcohol consumption has complex effects on the immune system; chronic and excessive consumption can lead to immunosuppression, affecting various cells of the immune system, such as T-cells, B-cells, and natural killer cells [25]. This explained the lower concentrations of IgG antibodies recorded in alcoholics in our study. Similarly, tobacco use strongly impairs the ability of the immune system to defend the host against infections and can also affect immune cell function, thus reducing antibody responses to vaccines [26]. Therefore, lower concentrations of IgG antibodies against the SARS-CoV-2 spike protein observed in tobacco users in our study might be attributed, at least partly, to the harmful effects of tobacco on the immune response.

The number of memory B-lymphocytes increased after the complete vaccination schedule, which might indicate an adaptive immune response, considering that these cells are responsible for producing antibodies in the long term after re-exposure to the virus [17]. In this context, the immunophenotyping results showed an increase in T-lymphocytes (CD4+ and CD4 T memory cells), B memory cells, classical monocytes, regulatory T-cells, and natural killer cells. In a non-indigenous population in Brazil, after the first dose of the CoronaVac vaccine, a substantial increase in the subpopulations of the CD4+ and CD19+ T-cells associated with the phenotypic profiles of immunological memory [18] were observed. Also, some multicenter studies showed that the immune response was higher after the second dose of the CoronaVac vaccine [19,20,21]. Vaccination with CoronaVac in this indigenous community elicited robust cellular and humoral immune responses, similar to those previously reported in non-indigenous populations [20,27,28]. These findings highlight the significance of the two-dose regimen in establishing an effective immune response.

In our cohort study, an increase in immune cell counts, including those of lymphocytes and monocytes, was measured after the complete vaccination schedule of the CoronaVac vaccine. These findings were also confirmed through the identification of subpopulations that had high levels of monocytes, CD19+ lymphocytes, CD8+ T-lymphocytes, and CD4+ T-lymphocytes [23]. The increase in monocytes, specifically those exhibiting an activated M1 effector phenotype (CD14/CD16/HLA-DR), played a key role in mucosal protection, antiviral mechanisms, and the antigen presentation process in response to COVID-19. We determined these changes through immunophenotyping of these cells in indigenous populations, which was previously undocumented. These results showed that long-lasting immunological memory developed against SARS-CoV-2, which is a promising indicator of protection. These findings are important for making healthcare decisions involving indigenous populations, which often have many difficulties, such as challenges in accessing health services and greater susceptibility to infectious diseases [19,29].

Studies demonstrate the effectiveness of CoronaVac not only in preventing serious cases of COVID-19, but also in substantially reducing hospitalization and mortality rates associated with the disease. The robustness of the cellular immune response induced by CoronaVac appears as a crucial factor in this success, characterized by an increase in the production of T-cells, including CD4+ and CD8+ T-lymphocytes. These T-cells play a crucial role in identifying and eliminating cells infected by the SARS-CoV-2 virus, thus strengthening the body’s defense mechanisms against the virus [30,31,32].

The response to the CoronaVac vaccine has shown promise in containing the spread of the coronavirus. Studies and data indicate the effectiveness of the vaccine, not only in preventing serious cases and hospitalizations, but also in significantly reducing transmission of the virus [33,34]. This multifaceted impact on individual and population health outcomes underscores the integral role of the vaccine in broader efforts to control the ongoing pandemic. Collective evidence from studies highlights the importance of CoronaVac in mitigating the severity of COVID-19, reducing mortality rates, and actively contributing to the overall containment of viral spread, thus reinforcing its position as a key tool in global public health strategies [21,34,35]. However, the unavailability of monitoring the population at a third time point made it impossible to carry out specific observations or analyses to assess the incidence of SARS-CoV-2 infections after vaccine administration in our study.

In addition, CoronaVac’s response stands out for the speed with which it was developed and made available to the population. The large-scale production capacity and ease of storage make this vaccine a valuable tool in vaccination campaigns around the world [36]. The positive response seen thus far reinforces confidence in inactivated vaccines and underscores the continued importance of global cooperation in vaccine research, development, and distribution to meet the persistent challenges posed by the pandemic [14].

Vaccination of indigenous people in Brazil plays a crucial role in mitigating the impacts of the COVID-19 pandemic and protecting these vulnerable communities [37]. Therefore, protecting these communities against COVID-19 also preserves traditions, languages, and knowledge transmitted over generations, since the health of these communities is intrinsically linked to their cultural resilience, and vaccination plays a vital role in safeguarding this patrimony [38]. Our findings might encourage further evaluation of the efficacy of other vaccines in indigenous populations, durability, and specificity of immune response, as well as potential barriers to the access and uptake of vaccines. Also, our study may provide a promise for targeted public health policies of this communities.

Our study had some limitations. First, the sample size of our indigenous population was small, which might limit the generalizability of our findings to larger indigenous communities. Second, as this was an observational study, we could not establish causality between lifestyle factors (alcohol and tobacco use) and the observed immune response. Also, the lack of a control group, and the diversity in the indigenous populations studied, may have affected the interpretation of our findings. The duration of four months might not capture the long-term durability of the immune response following vaccination. It is important to highlight that the immune response identified in this population may also be influenced by a variety of other antigens in the interim. Thus, the differences may be attributed to alternative factors; however, it is difficult to consider and incorporate them into the statistical analyses. Therefore, longer-term follow-up studies are necessary to better understand the sustained efficacy of the vaccine in this population. Despite that, to our knowledge this is the first study to report the immune response induced by COVID-19 vaccines in indigenous populations.

## 5. Conclusions

These results highlight that vaccination with CoronaVac in this community was able to elicit cellular and humoral immune responses, similar to those previously reported in non-indigenous populations. These findings underscore the significance of the two-dose regimen in establishing an effective immune response. Additionally, they emphasize the crucial importance of vaccination in health promotion, highlighting the effectiveness of inclusive strategies that address diverse demographic needs while overcoming economic barriers.

## Figures and Tables

**Table 1:** Sociodemographic characteristics and IgG positivity profile of anti-spike IgG QuantiVac™ ELISA assay of participants in this study.

	Participants (N = 91)	%	Anti-Spike IgG-^+^Positive (N = 61)	%	OR	95% CI	Χ² (*p)*
**Village**							
Bororó	42	45.65	33	78.57	2.655	1.051 to 6.707	**0.038**
Jaguapiru	49	53.26	29	59.18			
**Ethnicity**							
Guarani	24	26.09	18	75.00	4.134	0.834 to 2.786	0.170
Kaiowá	50	54.35	35	70.00			
Terena	18	19.57	9	50.00			
**Gender**							
Female	72	78.26	52	72.22	2.228	0.7930 to 6.263	0.128
Male	19	20.65	10	52.63			
**Age**							
18–29	44	47.83	29	65.91	0.937	0.392 to 2.242	0.884
≥30	47	51.09	32	68.09			
Governmental beneficiary **							
Yes	65	70.65	46	70.77	1.664	0.653 to 4.241	0.285
No	26	28.26	15	57.69			
**Education**							
<1 year	9	9.78	6	66.67	0.7347	0.166 to 3.251	0.684
1–8 years	65	70.65	48	73.85			
>9 Years	15	16.30	7	46.67	2.285	0.410 to 12.732	0.345
**Working**							
Yes	21	22.83	14	66.67	1.048	0.375 to 2.927	0.927
No	70	76.09	47	67.14			
**Family income**							
up to USD 250	43	46.74	27	62.79	0.583	0.237 to 1.430	0.238
USD 250–USD 500	48	52.17	36	75.00			
**Residents per house**							
<3 People	9	9.78	6	66.67	0.894	0.199 to 4.007	0.884
4–5 People	54	58.70	37	68.52			
>5 People	28	30.43	18	64.29	1.111	0.227 to 5.432	0.896
**Influenza Vaccine**							
Yes	85	92.39	60	70.59	12.200	1.355 to 109.769	**0.025**
No	6	6.52	1	16.67			
Use Tobacco ***							
Yes	30	32.61	10	33.33	0.096	0.034 to 0.265	**<0.001**
No	61	66.30	51	83.61			
Drink Alcohol ***							
Yes	18	19.57	1	5.56	0.012	0.001 to 0.102	**<0.001**
No	73	79.35	60	82.19			
**Contact with COVID-19 cases**							
Yes	32	34.78	20	62.50	0.714	0.289 to 1.763	0.465
No	61	66.30	41	67.21			
**COVID-19 symptoms prior to this study**							
Yes	40	43.48	26	65.00	0.947	0.492 to 1.823	0.163
No	51	55.43	35	68.63			

^+^ Positive on Anti-SARS-CoV-2 QuantiVac™ IgG ELISA assay after being vaccinated. OR: odds ratio; 95% CI: 95% confidence interval; ** Participants dependent on government financial assistance; *** Participants self-reporting continuous alcohol consumption and regular smoking. For the multinomial variables Ethnicity, Schooling, and Residents per household, the OR was calculated based on the reference category (category in which the OR and *p*-value appear). The significance level adopted for all analyses was set at <0.05. The *p*-values were obtained after Dunn’s correction.

## Data Availability

Data are contained within the article.

## Supplementary Materials

The following supporting information can be downloaded at: https://www.mdpi.com/article/10.3390/vaccines12040402/s1. Supplementary Table S1. Immune response of individuals previously exposed to the SARS-CoV-2. The before- and after-vaccination immune response metrics of individuals previously exposed to SARS-CoV-2 were used. The 95% CI refers to the 95% confidence interval. The significance level adopted for all analyses was set at <0.05. The *p*-values were obtained after Dunn’s correction.
