# Supplementary material for: Immune Response to an Inactivated Vaccine of SARS-CoV-2 (CoronaVac) in an Indigenous Brazilian Population: A Cohort Study
Source: Vaccines (Basel). 2024 Apr 10;12(4):402. doi: 10.3390/vaccines12040402 (PMC11053429; doi:10.3390/vaccines12040402)
Supplement: Supplementary file 1 [file vaccines-12-00402-s001.zip › vaccines-2798198-supplementary.pdf]

Supplementary table 1. Evaluation of the immune response of individuals previously exposed to the virus.

**Cellular Immune Response**	**95% CI**	***P* Value**	**Adjusted *P Value****
T CD4+ lymphocytes	65.97 (64.84 - 67.09)	0.0020*	0.0187*
T CD8+ lymphocytes	20.45 (19.37 - 21.52)	0.3510	1.0000
T regulatory cells	48.56 (46.89 - 50.22)	0.4300	1.0000
T lymphocytes	63.29 (62.07 - 64.51)	0.6480	1.0000
T CD4 memory cells	48.56 (46.89 - 50.22)	0.6700	1.0000
B memory cells	4.36 (3.89 - 4.84)	0.4680	1.0000
Non-classical monocyte	7.20 (6.01 - 8.39)	0.4830	1.0000
Intermediate monocyte	5.84 (4.99 - 6.69)	0.5750	1.0000
Classical monocytes	7.70 (6.98 - 8.44)	0.9610	1.0000
B lymphocytes	11.74 (10.79 - 12.69)	0.6010	1.0000
Natural killer cells	17.66 (16.73 - 18.60)	0.7160	1.0000

The pre and post-vaccination immune response metrics of individuals previously exposed to SARS-CoV-2 were used. 95% CI: 95% confidence interval. The significance level adopted for all analyses was set at <0.05. The p-values were obtained after Dunn's correction.
